# Biosynthesis of the active compounds of *Isatis indigotica* based on transcriptome sequencing and metabolites profiling

**DOI:** 10.1186/1471-2164-14-857

**Published:** 2013-12-05

**Authors:** Junfeng Chen, Xin Dong, Qing Li, Xun Zhou, Shouhong Gao, Ruibing Chen, Lianna Sun, Lei Zhang, Wansheng Chen

**Affiliations:** 1 Department of Pharmacy, Changzheng Hospital, Second Military Medical University, Shanghai 200003, China; 2 Department of Pharmaceutical Botany, School of Pharmacy, Second Military Medical University, Shanghai 200433, China; 3 Analysis and Testing Center, School of Pharmacy, Second Military Medical University, Shanghai 200433, China; 4 Department of Pharmacognosy, School of Pharmacy, Second Military Medical University, Shanghai 200433, China

**Keywords:** *Isatis indigotica*, Transcriptome sequencing, Biosynthetic pathways, Metabolite profile, Co-expression analysis

## Abstract

**Backgroud:**

*Isatis indigotica* is a widely used herb for the clinical treatment of colds, fever, and influenza in Traditional Chinese Medicine (TCM). Various structural classes of compounds have been identified as effective ingredients. However, little is known at genetics level about these active metabolites. In the present study, we performed *de novo* transcriptome sequencing for the first time to produce a comprehensive dataset of *I. indigotica*.

**Results:**

A database of 36,367 unigenes (average length = 1,115.67 bases) was generated by performing transcriptome sequencing. Based on the gene annotation of the transcriptome, 104 unigenes were identified covering most of the catalytic steps in the general biosynthetic pathways of indole, terpenoid, and phenylpropanoid. Subsequently, the organ-specific expression patterns of the genes involved in these pathways, and their responses to methyl jasmonate (MeJA) induction, were investigated. Metabolites profile of effective phenylpropanoid showed accumulation pattern of secondary metabolites were mostly correlated with the transcription of their biosynthetic genes. According to the analysis of UDP-dependent glycosyltransferases (UGT) family, several flavonoids were indicated to exist in *I. indigotica* and further identified by metabolic profile using UPLC/Q-TOF. Moreover, applying transcriptome co-expression analysis, nine new, putative UGTs were suggested as flavonol glycosyltransferases and lignan glycosyltransferases.

**Conclusions:**

This database provides a pool of candidate genes involved in biosynthesis of effective metabolites in *I. indigotica*. Furthermore, the comprehensive analysis and characterization of the significant pathways are expected to give a better insight regarding the diversity of chemical composition, synthetic characteristics, and the regulatory mechanism which operate in this medical herb.

## Background

*Isatisindigotica* Fort. (Brassicaceae) is a biennial herbaceous plant used as an important and popular herbal medicine in TCM with a long history. *Isatidis Radix* (Banlangen, *Isatis* root) and *Isatidis Folium* (Daqingye, *Isatis* leaf) are widely used for antibacterial, antiviral, and immune regulatory effects in the treatment of colds, fever, and influenza [1-3], especially for the treatment of severe acute respiratory syndrome (SARS) and H1N1-influenza [4-6].

To date, numerous active compounds have been identified from *I. indigotica* and the related species *Isatis tinctoria*[7]. These compounds are mainly divided into three classes, namely, alkaloids, phenylpropanoids, and terpenoids as listed in the Additional file 1: Table S1. Among these ingredients, the pharmaceutical activities of the indole alkaloids are mostly reported. Indirubin, isaindigotone, 5-hydroxyoxindole, indole-3-carboxaldehyde, and trytanthrin are validated to be the active substances for the doxorubicin resistance, leukocyte inhibition, and antiviral activities [8-12]. Phenylpropanoids is another major group of active compounds, which is mainly comprised of lignans and flavonoids. Lignans, including pinoresinol, lariciresinol, and lignan glycoside derivatives as lariciresinol-4′-*bis*-*O*-β-D-glucopyranoside were identified as anti-inflammatory and antiviral constituents [13,14]. In addition, compounds such as sitosterols, epigitrin, epiprogoitrin, and hypoxanthine, etc. also are involved in the major drug actions of *I. indigotica*. However, most of these compounds are presented in *I. indigotica* at alow natural abundance, such as lariciresinol (0.24‰ DW), lariciresinol-4′-*bis*-*O*-β-D-glucopyranoside (0.4‰ DW), and hypoxanthine (0.49‰ DW) [13,15]. Therefore, increasing the content of the active metabolites is of significance for enhancing the quality of *I. indigotica*, and also meets the growing market requirements.

Metabolic engineering approaches have emerged as a very powerful tool for increasing the production of valuable compounds in plants. With in-depth understanding of biosynthesis, the content of valuable compounds is dramatically increased by bio-engineering techniques in many medicinal plants as *Catharanthus roseus*[16], *Hyoscyamus niger*[17], and *Salvia miltiorrhiza*[18]. However, because of the limited information of biosynthesis pathways, there has been no successful progress on metabolic engineering in *I. indigotica* until now. Therefore, a deep understanding of the biosynthetic pathways of the various compounds produced by the plant becomes the first imperative. As a non-model plant species, little information was initially available to achieve this goal. In previous studies, the genes involved in lignan synthesis were isolated and characterized, including *PAL* (DQ115905), *C4H* (GU014562), *4CL* (GU937875), *CCR* (GQ872418), *CAD* (GU937874), *C3H* (JF826963), *CCoAOMT* (DQ115904), and *PLR* (JF264893) by homologous cloning [19,20]. Nevertheless, the slow process of homologous cloning has afforded only limited progress toward a complete understanding of these diverse, biosynthetic pathways in *I. indigotica*. Most of genes involved in secondary metabolites synthesis and the corresponding regulatory genes for these active compounds still remain unclear.

To obtain a general database of genes, 454 RNA deep sequencing was employed in order to evaluate the transcriptome of *I. indigotica*. By this *de novo* technique, it was possible to identify a set of putative genes involved in the pathways of secondary metabolism, especially those genes related to the biosynthesis of the valuable active compounds. The aim in this research was to establish a candidate gene pool of *I. indigotica*, and to assist in the discovery of new genes related to the secondary metabolic pathways. Meanwhile, metabolite analysis was carried out following the indications offered by the transcriptome. Integrated analysis of the transcriptome and the secondary metabolites will lead to an in-depth knowledge of both the pool of metabolites and biosynthetic processes for the formation of the active compounds in *I. indigotica*.

## Methods

### Plant materials and induction

The plant of *I. indigotica* was grown in the medicinal plant garden of the Second Military Medical University, Shanghai, China, and was identified by Professor Hanming Zhang. The organs of flowering plantlets, including flowers, leaves, stems, and roots were collected, respectively in April, and frozen immediately in liquid nitrogen for storage at −80°C. The *I. indigotica* hairy root cultures were maintained and sub-cultured in this laboratory. The hairy root material was cultured in 200 mL of 1/2 B5 liquid medium at pH 5.6. After 3–4 weeks of shaking culture, the hairy roots at the exponential phase were prepared for induction. A sample of 0.5 μM of MeJA (Sigma, USA) dissolved in ethanol was added to 200 mL of 1/2 B5 liquid medium for the induction. Solvent at the same volume was added into the control group. Hairy root cultures were collected at 0 h, 12 h, and 24 h after treatment, respectively. Samples were frozen and stored in liquid nitrogen until examination.

### RNA isolation and sequencing

Total RNAs were isolated with TRIzol reagent (Invitrogen, USA) according to manufacturer’s protocol. mRNA was purified from total RNA using the Oligotex mRNA Midi Kit (Qiagen, Germany). For 454 sequencing, the RNA extractions from different organs were mixed to a total amount of 20 μg. RNA of *I. indigotica* hairy roots was extracted for Solexa sequencing. A whole-plate sequencing run was performed with 454 Roche GS FLX platform. Paired-ends Solexa sequencing producing10 million reads per sample was carried out on Illumina HiSeq2000 platform. All sequencings were purchased from the Shanghai Majorbio Bio-pharm Technology Corporation.

### *De novo* assembly and functional annotation

After sequencing, the raw sequence data were first purified by trimming adapter sequences and removing low-quality sequences. The combined assembling of reads obtained by 454 and Solexa sequencing was subjected to Trinity (http://trinityrnaseq.sourceforge.net). Readswere combined with overlap of certain length to produce longer contigs (unigenes). The assembly was conducted using the default parameters. Reads that did not fit into a contig were defined as singletons. The resulting singletons and unigenes represented the *I. indigotica* candidate gene set. After assembling, BLASTx alignment (*e* value <1.00E - 5) of all-unigenes against protein databases, including the NCBI non-redundant(Nr) protein database, Swiss-Prot protein database, Kyoto Encyclopedia of Genes and Genomes (KEGG) pathway database, and the Cluster of Orthologous Groups (COG) database. The next step was to retrieve the proteins that had the highest sequence similarity with the obtained unigenes and determine their functional annotations.

### Quantitative real-time reverse transcription-PCR

A sample of 1 μg of total RNA was reverse-transcribed by Superscript III Reverse Transcriptase (Invitrogen, USA). The PCRs were performed according to the instructions of the SYBR premix Ex Taq kit, and carried out in triplicate using the TP8000 real-time PCR detection system (TaKaRa, China). Gene-specific primers were designed by Primer3 (http://frodo.wi.mit.edu/primer3/). The primers for multiple gene families were designed to avoid homology regions by homology alignment. The length of the amplicons was between 250 bp and 350 bp. The primer sequences are listed in the Additional file 2. Housekeeping gene *IiPOLYUBIQUITIN1* was selected as the internal reference. Thermo cycler conditions comprised an initial holding at 50°C for 120 sec and then at 95°C for 10 min. This step was followed by a two-step SYBRPCR program consisting of 95°C for 15 sec and 60°C for 60 sec for 40 cycles. Standard deviations were calculated from three PCR replicates. The specificity of amplification was assessed by dissociation curve analysis, and the relative abundance of genes was determined using the comparative Ct method.

### Metabolites analysis of MeJA treated *I. indigotica* hairy roots

*I. indigotica* hairy roots sample (50 mg) was freeze dried at 40°C and ground into powder. Subsequently, sample was extracted with 80% methanol under sonication for 30 min for twice. The extraction was diluted to 50 mL total volume, and then filtered through a 0.2-μm organic membrane and store at -20°C for analysis. The concentration of the metabolites was determined by triple-quadrupole mass spectrometer (Agilent 6410, Agilent, Santa Clara, CA) equipped with a pump (Agilent 1200 G1311A, Agilent) and an autosampler (Agilent G1329A, Agilent). Chromatography separation was performed with Agilent ZORBAS SB-C18 column (100 mm × 2.1 mm, 3.5 μm particle size). A mobile phase consisting of acetonitrile: methanol (5: 95, v/v) was used, with the flow rate set at 0.3 ml min^−1^ and a 5 min run time. Multiple reactions monitoring (MRM) mode was used for the quantification and the selected transitions of *m/z* were 401 → 110 for coniferin, 359 → 329 for lariciresinol, 361 → 164 for secoisolariciresinol, 357 → 164 for mataireisnol, 357 → 151 for pinoresinol, 179 → 146 for coniferyl alcohol, 685 → 523 for secoisolariciresinol diglucoside, 286 → 117 for kaempferol, 302 → 151 for quercetin, and 316 → 299 for isorhamnetin. Standards of lariciresinol, and pinoresinol were prepared in our laboratory, other standards were purchased from Sigma-Aldrich (St. Louis, MO).

### Q-TOF LC/MS analysis of flavonoids

Roots and leaves were harvested from plantlets of *I. indigotica*. Samples were dried at 40°C to constant weight and powdered for extraction. A powdered sample (200 mg) was extracted in solvent (methanol: H_2_O = 8:2) by reflux extraction method at 80°C three times, and concentrated to 50 mL. Chemical analysis was performed using an ultra-performance liquid chromatography system (UPLC, Agilent 1290, Agilent Technologies, Waldbronn, Germany) fitted with an Agilent 6538 UHD Accurate-Mass Q-TOF LC/MS (MS-TOF, Agilent Technologies, Santa Clara, CA, USA) equipped with an ESI interface. The chromatographic separation of compounds was achieved using an Agilent Eclipse Plus C18 column (2.1 mM × 100 mM,1.8 μM) in binary gradient mode at a flow rate of 0.3 mL/min. Column oven and auto sampler temperatures were maintained at 40°C and 4°C, respectively. The column temperature was held at 25°C and the sample injection volume was 5 μL. The full scan mass spectra were measured in a scan range from 100 to 1,500 amu with a scan resolution of 13,000 *m*/*z*/s. Spectra were acquired in the positive and negative ionization modes. Data analysis was performed using the Agilent Mass Hunter Workstation software. The target compounds were identified by the product ion spectrum (MS^2^), in the positive and negative ion modes. The extracted fragment mass ions of the target compounds were as follows: kaempferol: *m/z* 287.055 ([M + H] ^+^); 285.041 ([M-H]^-^). quercetin: *m/z* 303.049 ([M + H]^+^); 301.036 ([M-H]^-^). kaempferol-3-*O*-glucoside: *m/z* 449.108 ([M + H]^+^); 447.0963 ([M-H]^-^). quercetin-3-*O*-rhamnoside-7-*O*-rhamnoside: *m/z* 595.166 ([M + H]^+^); 593.154 ([M-H]^-^). kaempferol-3-*O*-rhamnoside-7-*O*-glucoside: *m/z* 595.166 ([M + H]^+^); 593.154 ([M-H]^-^). quercetin-3-O-glucoside-7-*O*-rhamnoside: *m/z* 611.16 ([M + H]^+^); 609.148 ([M-H]^-^). quercetin-3-*O*-rhamnoside-7-*O*-glucoside: *m/z* 611.16 ([M + H]^+^); 609.148 ([M-H]^-^).

### Co-expression analysis

Co-expression analyses were performed using a co-expression Gene Search algorithm on the RIKEN PRIMe website (http://prime.psc.riken.jp/). A total of 71 *Arabidopsis* genes with the highest homology to *I. indigotica UGT*s were collected as query genes (Additional file 3). The co-expression relationships of the genes exhibited correlation coefficients > 0.525 with the query genes, the co-expression graph was depicted by the Pajek program (http://vlado.fmf.uni-lj.si/pub/networks/pajek/).

### Phylogenetic analysis

Phylogenetic relationships were analyzed using MEGA version 5.5 [21]. The Poisson correction parameter and pair wise deletions of gaps were applied. The reliability of branching was assessed by the bootstrap re-sampling method using 1000 bootstrap replications.

## Results

### Transcriptome sequencing of Isatis indigotica

**Figure 1:**
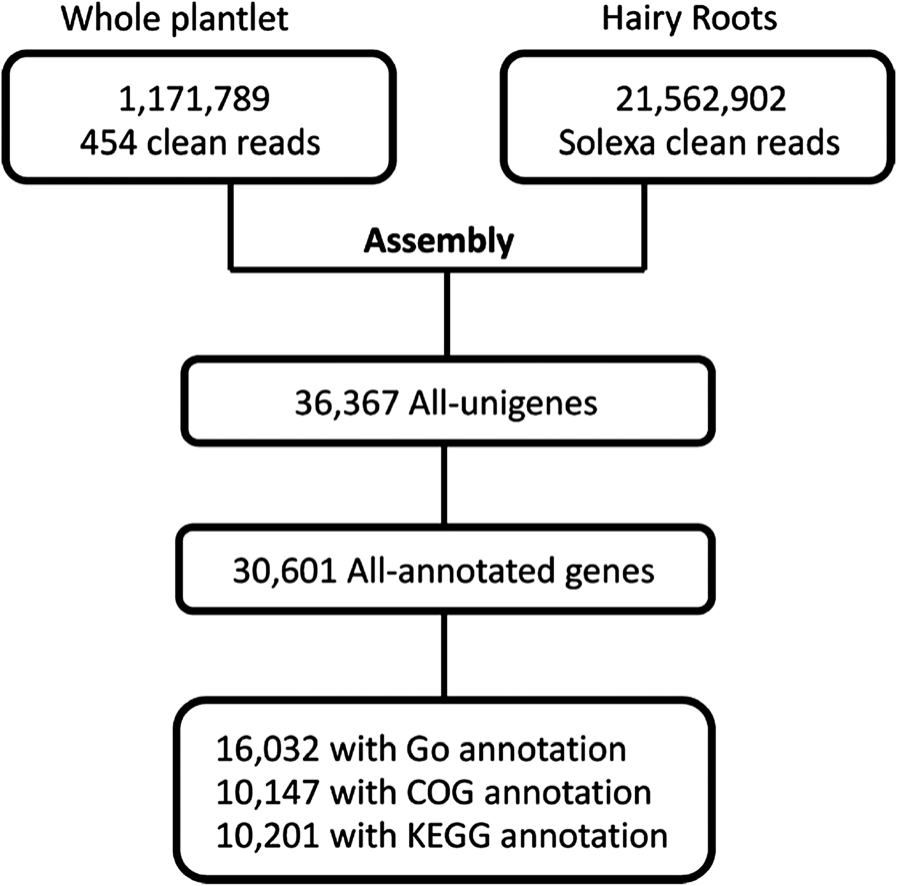
**Flowcharts of sequencing and annotation for *I. indigotica *transcriptome.** The steps include sequence assembly, GO annotation, COG annotation, and KEGG annotation.

The 454 pyrosequencing technology (Roche GS FLX) was employed to sequence the transcriptome of *I. indigotica*. To achieve maximized abundance of contigs, total RNA from *I. indigotica* roots, stems, leaves, and flowers was extracted, respectively, and then mixed. A normalized cDNA library was constructed with 20 μg of total RNA, and then was sequenced using the 454 pyrosequencing technology. A completely sequenced run produced 1,171,789 reads with an average length of 316 bp. In order to maximize the sequence diversity, an additional paired-end Solexa sequencing (Illumina HiSeq2000) was employed. This process afforded 21,562,902 reads in length of 101 bp (Figure 1). The 454 and Solexa reads were combined for assembly with Trinity (http://trinityrnaseq.sourceforge.net). After assembly, 36,367 unigenes with an average length of 1,115.67 bp were generated (Additional file 4). Compared with assembly only using the 454 reads, the number and the average length of the unigenes were significantly promoted by 117.42% and 58.69% (Additional file 5: Table S2), respectively. As a result, when the ORF prediction was carried out on the website of Trinity, 30,600 unigenes were predicted to contain an ORF region.

### Functional annotation of *I. indigotica* transcriptome

Annotation of the transcriptome was carried out to generate a transcriptome database of *I. indigotica*. A total of 30,600 ORFs were aligned to public protein databases by Blastp. Alignment of unigenes (5,767) without ORF predictions were subjected to Blastx. The unigenes were searched against the public databases (Nr, Nt, STRING, and KEGG). Finally, a total of 30,601unigenes were annotated in this manner. To further demonstrate the functional distribution of all unigenes, GO, COG, and KEGG analysis were subjected for function prediction and classification. As a result, a total of 16,032 unigenes were mapped to GO terms. The assignments were given to biological processes (34.8%), molecular functions (50.6%), and cellular components (14.6%) (Additional file 6). Among all of the GO terms, the vast majority were related to cell components (11,511), binding (9,957), cellular process (9,802), and metabolic processes (9,224). In addition, all of the unigenes were mapped into the records of the COG database and COG annotations were retrieved. Overall, 12,680 putative proteins were functionally classified into at least 25 protein families (Additional file 7). The cluster for general function prediction (29.65%) represented the largest group. Finally, the KEGG pathway analysis was performed to assign the biological pathways to the all of the unigenes. In total, 10,201 unigenes were assigned to 303 KEGG pathways (Additional file 8).

### Characterization and expression analysis of the genes involved in the putative indole alkaloid biosynthesis pathway

According to the GO analysis, 6% unigenes were assigned to secondary metabolite biosynthesis, in which the genes involved in the synthesis of the active compounds were included. According to the composition of the known active compounds in *I. indigotica*, the putative synthetic pathways of these compounds are described to present the synthetic characteristics and chemical composition of *I. indigotica*. The biosynthesis-related unigenes of indoles, terpenoids, and phenylpropanoids were identified from the current transcriptome assembly using computational approach.

**Figure 2:**
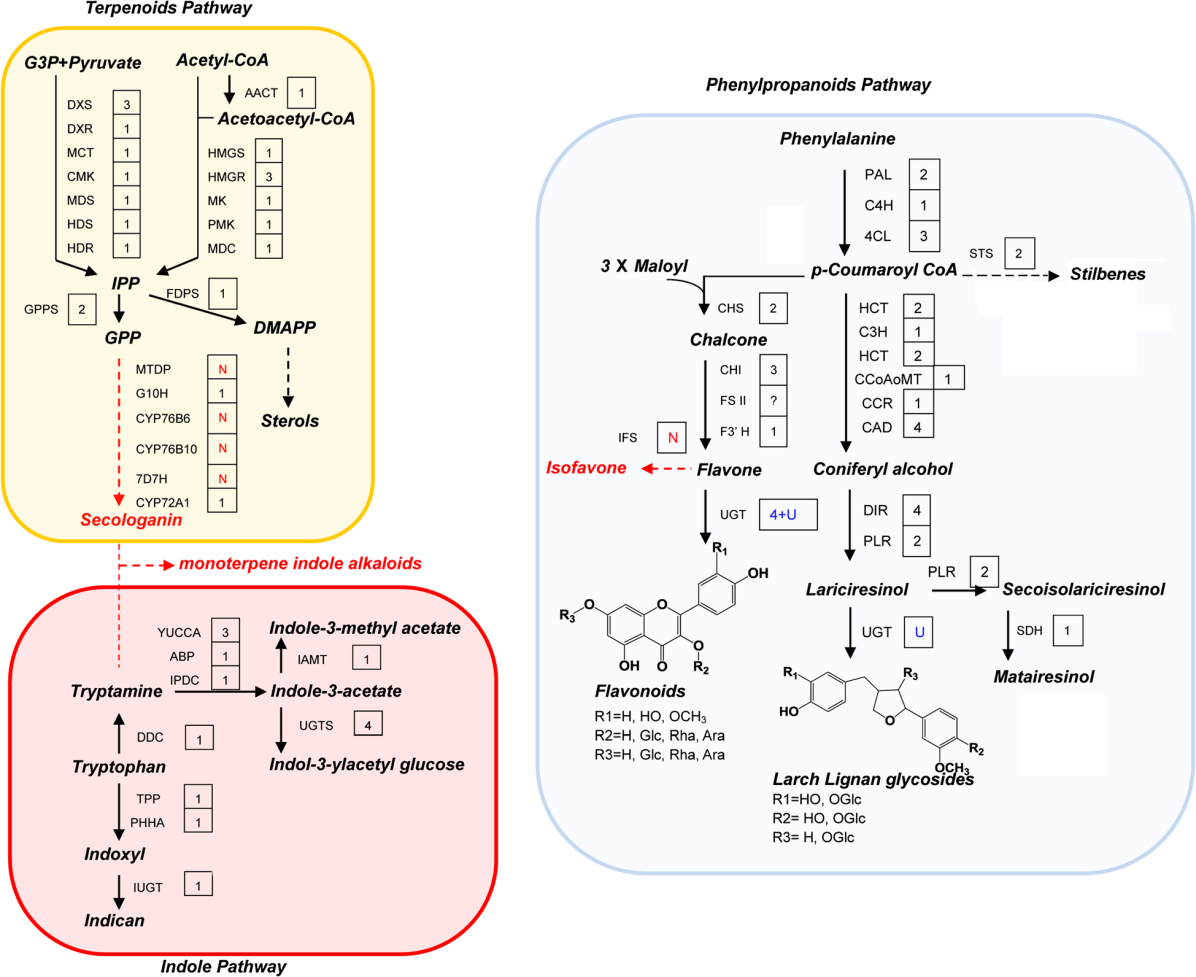
**Schematic for the putative biosynthetic pathways of three major classes of active compounds in *I. indigotica*.** Terponoids, indole derivatives, and phenylpropanoid pathways are marked with background in yellow, red, and blue, respectively. Red characters indicate the non-detected genes. Dashed line in red indicate undetected pathway. Figures in panes indicate the number of unigenes corresponding to the catalytic gene in the pathway; U, unknown; N, Not annotated.

Indole alkaloids, derived from the metabolism of tryptophan, are the most diverse alkaloids class in *I. indigotica* (Figure 2a). A total of 71 unigenes related to tryptophan metabolism were annotated, including 14 unigenes coding 9 enzymes which catalyzed the synthesis of indican, indole-3-acetate, indole-3-methylacetate, and indol-3-ylacetyl glucose. Although more than 40 indole derivatives have been reported in *I. indigotica*, the biosynthesis genes for most of them were unable to be identified due to the limited information of downstream biosynthetic pathways in plants.

**Figure 3:**
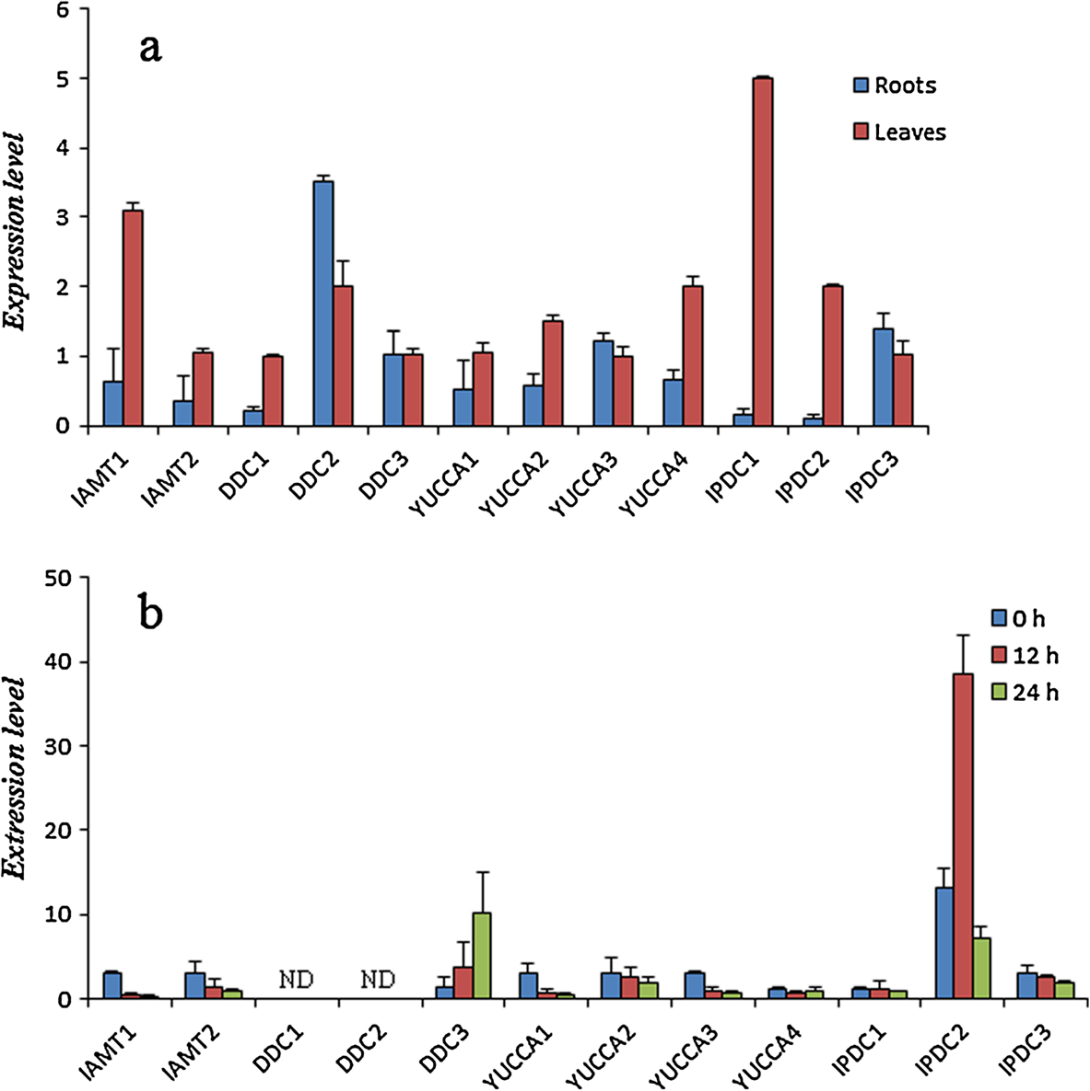
**Expression patterns of putative genes involved in the biosynthesis of indole alkaloids pathway. a**, organ-specific expression in roots and leaves of *I. indigotica*; **b**, expression pattern of indole biosynthesis genes in *I. indigotica* hairy roots treated with MeJA for 0 h, 12 h, and 24 h. Expression levels were quantified by qPCR. The level of each gene is relative to that of *UBIQUITIN* reference. Each data point is the average of three biological repeats. Error bars indicate SDs. N, None detected.

Expression characteristics of the synthetic pathway genes revealed the biosynthesis and accumulation patterns of catalysate. Organ-specific expression pattern of genes, including two methyl indole-3-acetate methyl transferase genes (*IAMT*), three aromatic amino acid decarboxylase genes (*DDC*), four YUCCA monooxygenase genes (*YUCCA*) and three indole pyruvate decarboxylase genes (*IPDC*) were examined (Figure 3a) by qRT-PCR. Expression levels of each gene in the roots and leaves were detected, in which the active compounds of *I. indigotica* were mostly accumulated. The results showed that most of the indole biosynthetic genes had superior expression levels in the leaves, suggesting that the leaves were the primary site for indole biosynthesis and accumulation. The indole precursor was reported to be synthesized and stored in the young leaves of *I. tinctoria*[22]. Thus, the expression characteristics of synthetic genes were indeed a reflection of the accumulation pattern of indole alkaloids.

MeJA is well-known for improving the accumulation of various secondary metabolites [23-25]. To investigate how the indole biosynthetic pathways respond to MeJA, the expression pattern of the relative genes was detected in MeJA-treated *I. indigotica* hairy roots (Figure 3b). The expression of most detected genes was depressed by MeJA. Only two unigenes *DDC3* and *IPDC2* were up-regulated. In addition, *YUCCA4* and *IPDC1* did not show obvious changes in transcription, *DDC1* and *DDC2* were not detected in *I. indigotica* hairy roots.

### Characterization and expression analysis of the genes involved in the putative terpenoid biosynthesis pathway

Sterols are the major effective terpenoids in *I. indigotica*. Their biosynthesis is initiated by the synthesis of isopentenyldiphosphate (IPP). A putative biosynthetic pathway of terpenoids in *I. indigotica* is shown in Figure 2b. In total, 54 unigenes relating to 20 enzymes leaded to synthesis of IPP and dimethylallyldiphosphate (DMAPP) were identified. Secologanin is the core structure of the terpenoid indole alkaloids [26]. However, several secologanin synthetic genes, including monoterpenyl-diphosphatase gene (*MTDP*), CYP76B8, CYP76B10, and 7-deoxyloganin 7-hydroxylase gene (*DL7H*), were not identified in the *I. indigotica* transcriptome. The result indicated the absence or low transcription level of monoterpenoids synthesis in *I. indigotica*.

**Figure 4:**
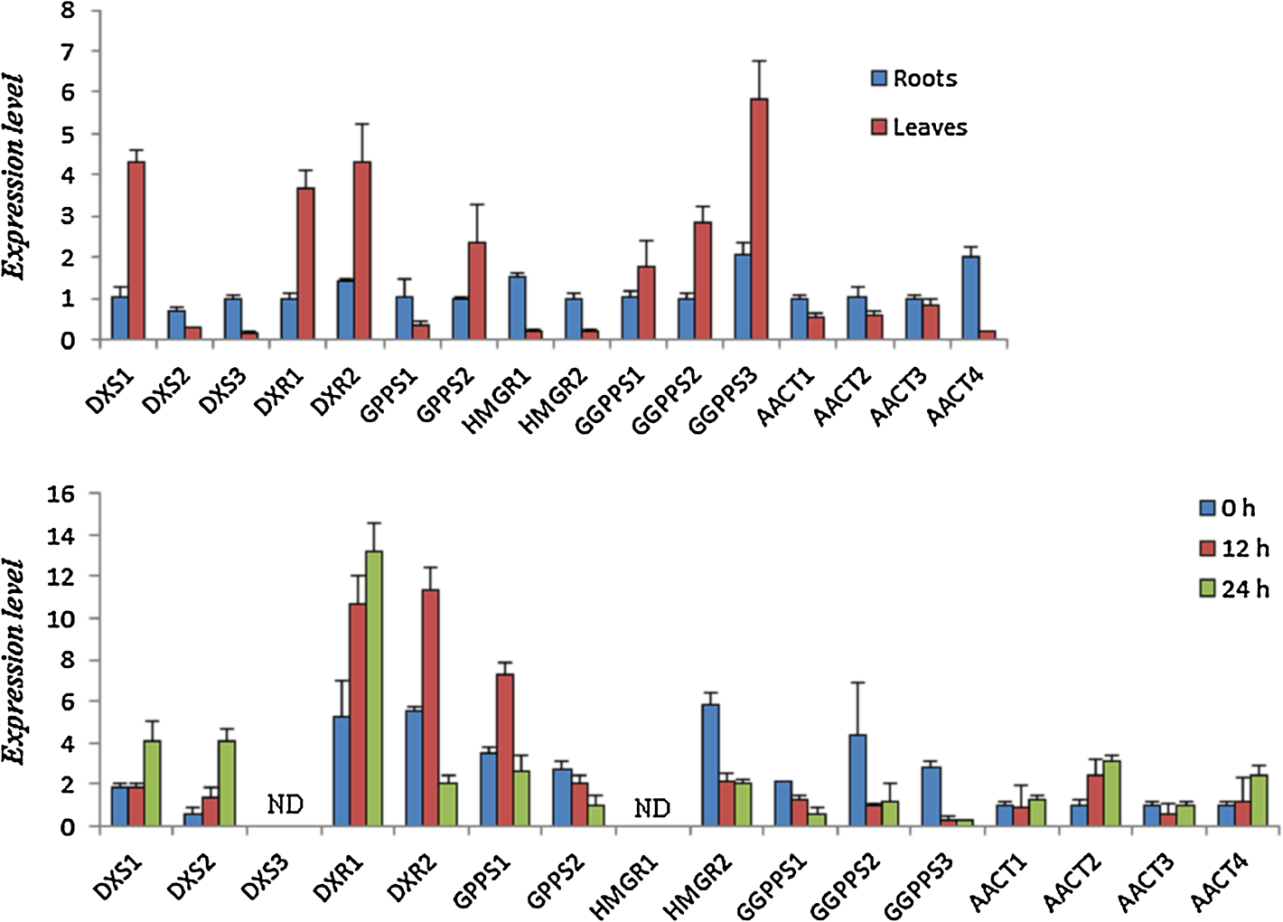
**Expression patterns of putative genes involved in the biosynthesis of terpenoids pathway. a**, organ-specific expression in roots and leaves of *I. indigotica*; **b**, expression pattern of terpenoids synthesis genes in *I. indigotica* hairy roots treated with MeJA for 0 h, 12 h and 24 h. The level of each gene is relative to that of *UBIQUITIN* reference. Each data point is the average of three biological repeats. Error bars indicate SDs. ND, None detected.

The organ-specific expression pattern of terpenoid related unigenes did not show obvious regularity. Significant differential expression pattern between members of 1-deoxy-D-xylulose 5-phosphate synthase (*DXS*), 1-deoxy-D-xylulose 5-phosphate reductoisomerase (*DXR*), geranylgeranyl diphosphate synthase (*GGPPS*), and acetyl-CoA *C*-acetyl transferase (*AACT*) multiple gene families was observed. *DXS1*, *DXR1*, *DXR2*, and three *GGPPS* unigenes showed higher expression levels in the leaves, whereas the remainder of the genes was all mainly expressed in the roots (Figure 4a). The results suggested that a complex biosynthesis and accumulation for different terpenoids in *I. indigotica*.

The expression of fifteen unigenes belonging to the *DXS*, *DXR*, *GGPS*, *GGPPS,* hydroxymethylglutaryl-CoA reductase gene (*HMGR*) and *AACT* families were detected under the induction of MeJA. These genes showed significantly different response patterns, except for *DXS*3 and *HMGR1* were undetectable in *I. indigotica* hairy roots (Figure 4b). Transcription of *DXS*s and *DXR*s were up-regulated, in contrast to the inhibited expression of *HMGR*2 and *GGPPS* genes. Two *GGPPS* genes responded to MeJA in the opposite pattern. *GGPPS1* was up regulated, while *GGPPS2* showed negative response.

### Characterization and expression analysis of the genes involved in the putative phenylpropanoid biosynthesis pathway

Lignans and flavonoids are the two major classes of phenylpropanoids in *I. indigotica*. A total of 35 unigenes, encoding up to 19 enzymes, were involved in the biosynthesis pathway for the lignans and flavonoids (Figure 2c). Moreover, composition of lignans and flavonoids are enriched by glycoslation catalyzed by multiple UGTs. However, so far only a few UGTs involved were designated a precise functional description in plant [27-29]. Four flavonoids and two lignan correlated UGTs were identified according to sequence identity with reported UGTs. It was noteworthy that two putative stilbene synthase genes (*STS*) were identified with e-value of 0 and 1.00E-131, respectively. Meanwhile, two unigenes also showed high similarity to chalcone synthase genes (*CHS*) with e-value of 0 and 1.00E-144. STS and CHS belong to the same family (type III polyketide synthases). Only a few residues are critical for their activity distinction [30,31]. Therefore, further investigations on function of two unigenes and stilbene metabolites were needed for the identification of these genes. Moreover, isoflavone synthase gene (IFS), which leads to the isoflavonoids synthesis, was not identified.

**Figure 5:**
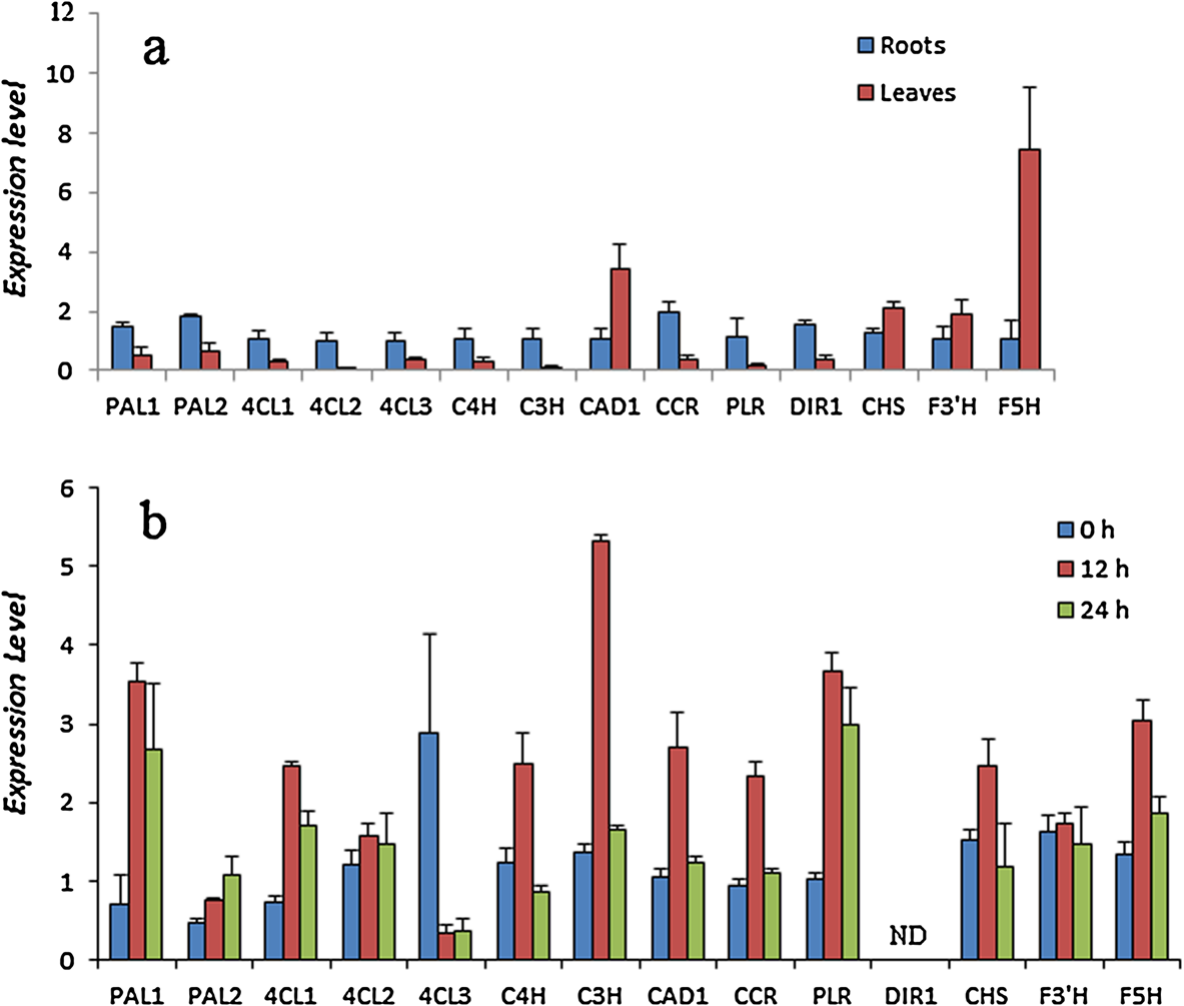
**Expression patterns of putative genes involved in the biosynthesis of phenylpropanoids pathway. a**, organ-specific expression in roots and leaves of *I. indigotica*; **b**, expression pattern of phenylpropanoids synthesis genes in *I. indigotica* hairy roots treated with MeJA for 0 h, 12 h and 24 h. The level of each gene is relative to that of *UBIQUITIN* reference. Each data point is the average of three biological repeats. Error bars indicate SDs. ND, None detected.

The expression pattern of fourteen unigens was examined (Figure 5). Most of them showed superior transcription levels in roots, except for *CHS*, *F3′H* and *F5H* which are involved in the flavonoid biosynthesis branch (Figure 5a). The results suggested higher accumulation of flavonoids in leaves. MeJA is reported to activate both the general and downstream aspects of the phenylpropanoid biosynthesis pathway in *Arabidopsis*[32,33]. To be consistent with *Arabidopsis*, the expression of most phenylpropanoid biosynthesis genes in *I. indigotica* was up-regulated by MeJA in different degree, except for *4CL3* (Figure 5b). The result suggested that MeJA induction could improve the accumulation of most of the phenylpropanoids in *I. indigotica*. Moreover, the transcript of *DIR1* was not detected in *I. indigotica* hairy roots.

### Accumulation of phenlypropanoids in *I. indigotica* under MeJA induction

**Figure 6:**
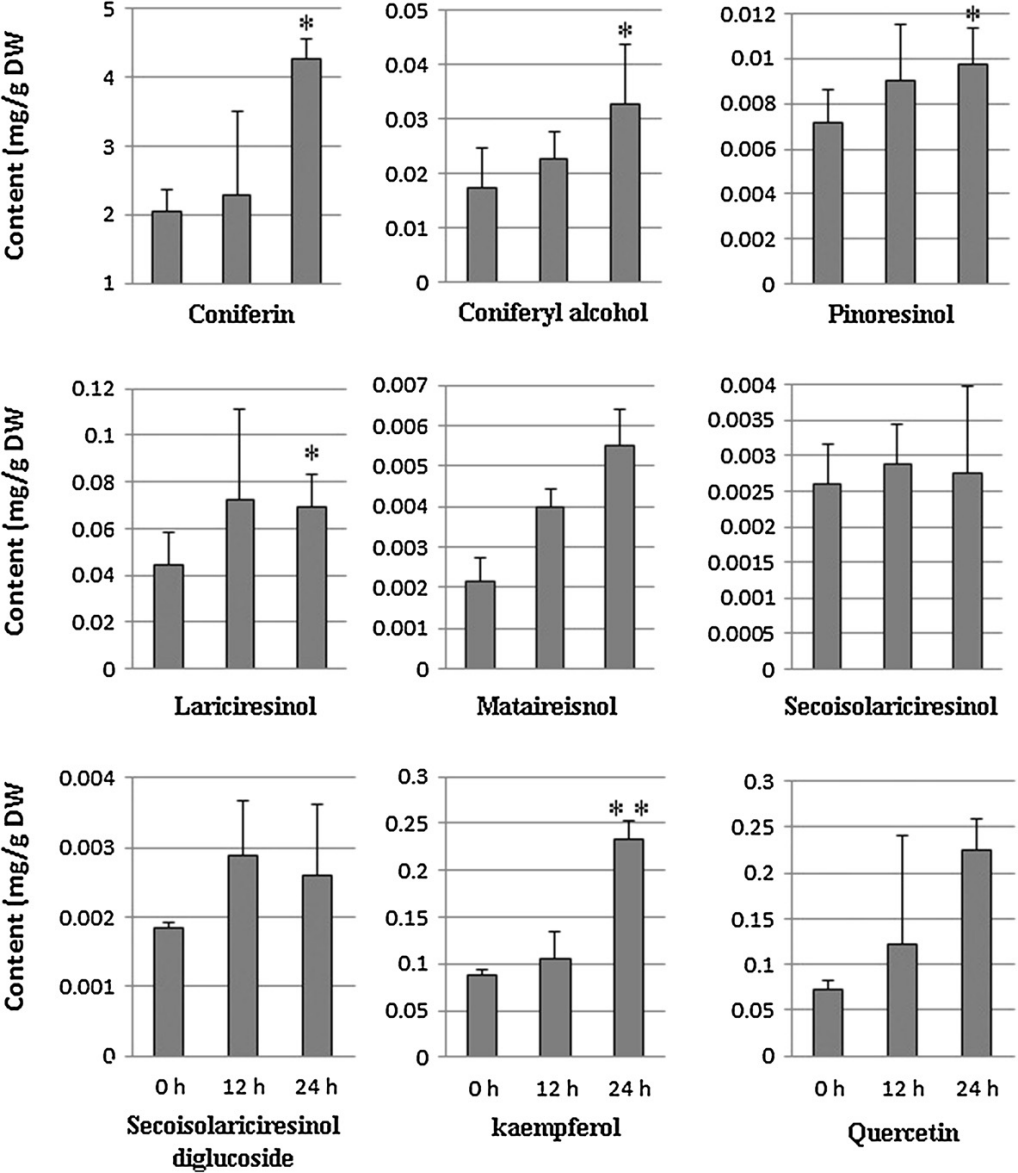
**MeJA-induced changes in the phenlypropenoids accumulation during *I. indigotica *hairy roots culture period.** The accumulation of metabolites in *I. indigotica* hairy roots after MeJA treatment for 12 h and 24 h was compared to control. Error bars show the standard deviations of independent strains. Asterisk indicates that difference is significant (*: p < 0.05, **: p < 0.01) compared with control.

As transcription level revealed, the accumulation of related metabolites would increase by MeJA induction. To discuss the correlation of MeJA induced transcription and metabolites, accumulation pattern of 10 phenylpropanoids including lignans and flavonoids (Additional file 9: Table S3) in MeJA treated *I. indigotica* hairy roots were examined using triple-quadrupole mass spectrometer. The content of target components after MeJA treatment for 12 h and 24 h was compared with control. The average content of three biological replicates was presented in Figure 6. Isorhamnetin was only detected in the control strain of hairy roots line 1 (Data not shown). Therefore, the content of isorhamnetion was not discussed here. As shown in Figure 6, all detected components showed increased accumulation at different level under MeJA induction. Accumulation of coniferin, coniferin alcohol, pinoresinol, lariciresinol, mataireisnol, and kaempferol increased significantly (*p*-value < 0.05) at 24 h after treatment. The result showed the phenlyopropanoids synthesis was activated by MeJA corresponding to the transcriptional variation, which indicated the accumaltion pattern of secondary products were mostly correlated with the transcription of their biosynthetic genes.

### UGTs indicate the flavonoid composition of *I. indigotica*

Among the synthetic genes, some specific gene families enrich the diversity of secondary metabolites, such as UGT family. UGTs catalyze transfer of the glycosyl group from nucleoside diphosphate-activated sugars (UDP-sugars) to a diverse array of secondary metabolites. Classes of effective compounds in *I. indigotica* like flavonoids and lignans are production of UGTs. As shown in Figure 2c, flavonoids synthetic genes, including flavonol synthase gene (*FLS*), flavonoid 3′- hydroxylase gene (*F3′H*), *O*-methyltransferase gene (*OMT*), were annotated. Meanwhile, flavonoids related UGTs (UGT78D1, UGT78D2, UGT73C6, and UGT89C1), were also identified [27-29], which suggested the synthesis of diverse flavonoids in *I. indigotica*. However, none of this class of flavonoids had ever been reported in *I. indigotica*. To validate these supposed flavonoids, mass spectrometric analysis was performed using UPLC-ESI-QTOF-MS. Profiles of the EIC (extracted ion chromatogram) are presented in Additional file 10. Table 1 showed extract mass, calculated molecular formula, retention time, and the putative flavonoids. As a result, six putative flavonol glycosides were identified in *I. indigotica*. The result indicated the existence of kaempferol derivatives in *I. indigotica*.

**Table 1:** Characteristics of putative flavonoids by UPLC-ESI-QTOF-MS

**Formula**	**Retention time (min)**	***m/z *** **(Positive)**	***m/z *** **(Negative)**	**Putative flavonoids**
C_21_H_20_O_11_	5.8	449.108 [M + H]^+^	447.096 [M-H]^-^	kaempferol-3-*O*-glucoside
C_27_H_30_O_14_	5.74	/	623.164 [M + HCOO]^-^	kaempferol-3-*O*-rhamnoside-7-*O*-rhamnoside
C_27_H_30_O_15_	4.93	595.166 [M + H]^+^	593.154 [M-H]^-^	kaempferol-3-*O*-rhamnoside-7-*O*-glucoside
C_27_H_30_O_15_	5.51	595.166 [M + H]^+^	593.154 [M-H]^-^	quercetin-3-*O*-rhamnoside-7-*O*-rhamnoside
C_27_H_30_O_16_	5.11	611.16 [M + H]^+^	609.148 [M-H]^-^	quercetin-3-*O*-glucoside-7-*O*-rhamnoside
C_27_H_30_O_16_	4.9	611.16 [M + H]^+^	609.148 [M-H]^-^	quercetin-3-*O*-rhamnoside-7-*O*-glucoside

### Distribution and co-expression analysis of UGTs in *I. indigotica*

As revealed above, UGTs play an important role in the diversity of plant secondary metabolites [34]. Besides the glycosylation reactions of flavonoids, glycosylation also occurs on different classes of natural products, such as indoles [35], lignans [36], and stilbenes [37]. In *I. indigotica*, a total of 147 UGTs were identified and classified into 41 families (Additional file 11). The largest UGT family was UGT76E which was comprised of 20 unigenes. The UGT72C and UGT75C families were not found in transcriptome of *I. indigotica*.

**Figure 7:**
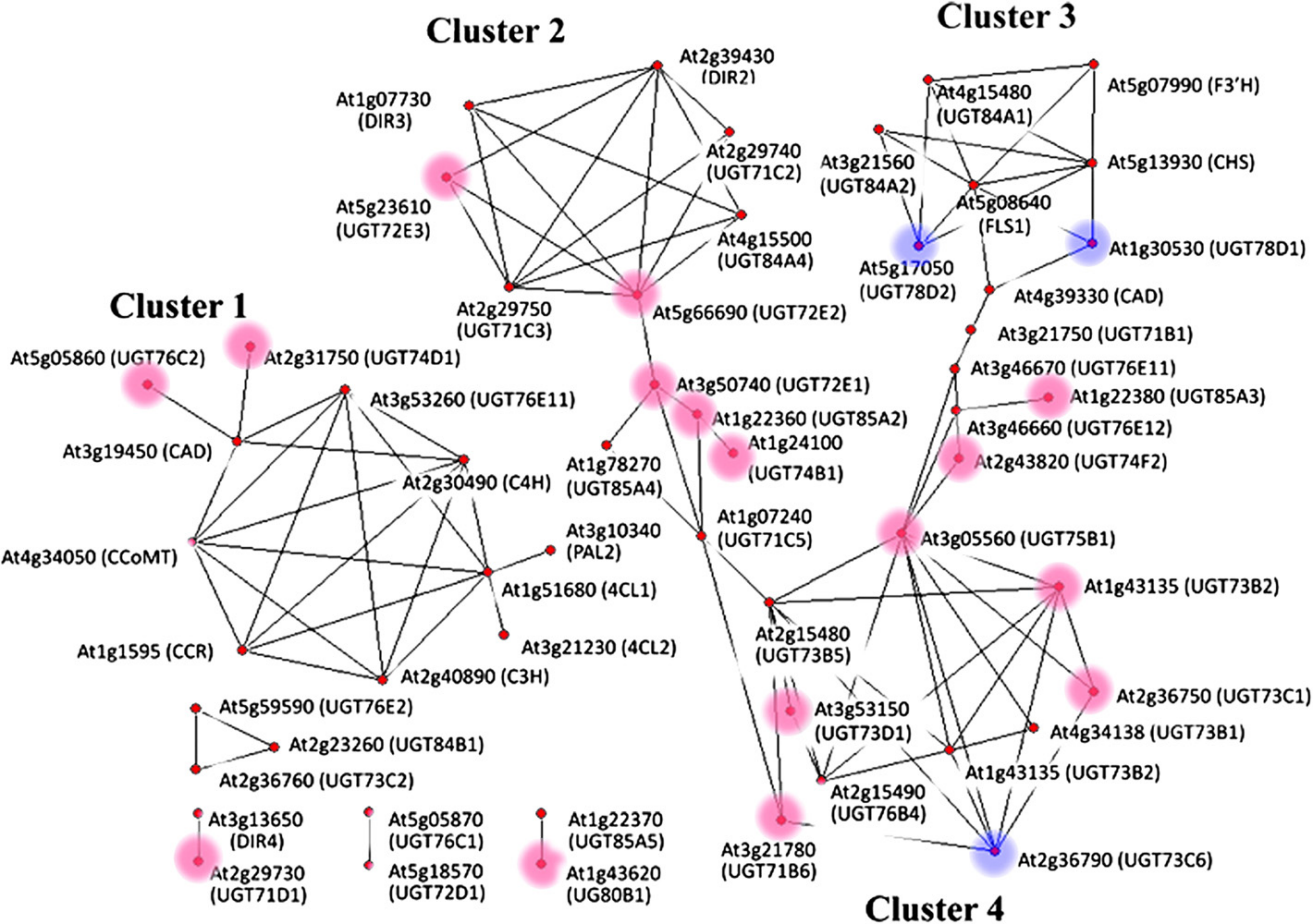
**Co-expression correlations of unigenes involved in the flavonoid and lignan biosynthesis**. Edges are drawn when the linear correlation coefficient is > 0.5. UGTs with detailed functional description are marked with blue circles; UGTs with functional prediction are marked with red circles.

By means of a correlation among the changes in transcriptional activity, gene function can be predicted. Co-expression analysis provides opportunities to explore the potential function of genes [38]. In order to screen the candidate UGTs involved in flavonoid and lignan biosynthesis in *I. indigotica*, transcriptome co-expression analysis according to expression profile of homologous *Arabidopisis* UGTs was performed*.* Homologous *Arabidopisis* genes, including 52 *UGT*s, ten flavonol synthesis genes, and nine lignan synthesis genes, were subjected as query. As shown in Figure 7, a total of 45 co-expressed genes showed greater correlation coefficient than 0.5 with at least one other genes, and were mainly classified into four major clusters. Cluster 1 was mainly made up of general phenylpropanoid biosynthesis genes, including *PAL*s, *4CL*s, and *C4H*, and lignan biosynthesis genes, such as *C3H*, *CCoAoMT*, *CAD*, and *CCR*. Cluster 2 and Cluster 3 contained flavonoid and lignan correlated genes, respectively. *DIR2* and *DIR3*, which located at downstream of lignan biosynthesis, were classified into cluster 2. In correspondence with *DIR2* and *DIR3*, five *UGT*s in cluster 2 were regarded as lignan glucosyltransferase genes. In cluster 3, flavone biosynthesis genes *CHS*, *F3′H*, and *FLS* were correlated with four *UGT*s. Besides *UGT78D1* and *UGT78D2*, which were known to be *O*-glucosytranferase genes, *UGT84A1* and *UGT84A2* were predicted to be flavonol glucosyltransferase genes due to the catalytic activity of correlative genes. In cluster 4, *UGT75B1* was predicted to be a flavonol glucosyltransferase gene based on the correlation with both cluster 3 and *UGT73C6*. Beside the major clusters, *UGT71D1* might be considered as a lignan glucosyltransferase gene according to the correlation with *DIR4*. In summary, five UGTs (71C1, 71C2, 71D1, 72E3, and 84A4) were predicted as lignan glucosyltransferases and four UGTs (73C1, 75B1, 84A1, and 84A2) were classified as flavone glucosyltransferases.

## Discussion

### The transcriptome sequencing of *I. indigotica*

With the increasing availability of second-generation sequencing, plant transcriptome sequences are appearing in increasing numbers. Due to the desire to understand the biosynthetic processes of bioactive compounds in *I. indigotica*, the complete transcriptome of *I. indigotica* was sequenced and analyzed. The technique of 454 RNA sequencing was employed to produce a database of expressed genes of *I. indigotica*. In order to obtain maximized coverage of the genes, a mixed RNA sample from different organs of the plant was used to construct a cDNA library. Meanwhile, additional Solexa sequencing was carried out to enrich the abundance isotigs. The results showed that the strategy was effective for maximization of the number and the length of the unigenes. Although there might be genes of low abundance or conditionally expressed genes absent in this database, this study presents the most abundant genetic resource concerning the important medicinal plant *I. indigotica.*

### Analysis of active compound synthesis according to the *I. indigotica* transcriptome

Indole alkaloids, flavonoids, and lignans are the three major classes of biologically active metabolites in *I. indigotica*. Based on the transcriptome annotation of *I. indgotica*, 104 unigenes coding 48 enzymes involved in indole, terpenoid, and phenylpropanoid biosynthesis were obtained, of which most are novel. The pool would provide candidate synthetic genes for further investigation of certain catalytic steps. Moreover, the expression patterns experiments of synthetic genes will generate an improved understanding of their functional characteristics. Several genes involved in each of the target pathways were encoded by multiple gene members with different organ specific expression patterns demonstrating the complexity of the biosynthesis of these classes of compounds in *I. indigotica*. The results supported the view that specific groups of phenylpropanoids [39] and terpenoids [40] were synthesized by specific metabolic channels organized by isoenzymes within the pathways. The distinctive responses to MeJA would provide useful information for improving production of effective components through genetic engineering. Interestingly, some transcripts with a high expression level in *I. indigotica* plantlet, such as *DDC2*, *DDC3*, *DX3*, *HMGR1*, and *DIR1*, were not detected in hairy root. The transcription of these genes might not happen in *I. indigotica* hairy roots or was in a very low level. The result indicated the specific characteristics of secondary metabolites in *I. indigotica* hairy roots.

The tanscriptome analysis not only make better understanding of secondary metabolites in *I. indigotica* on transcriptional level, but also provide useful information on its metabolites. Besides the biosynthesis genes related to the known compounds, the biosynthetic genes of non-reported compounds in *I. indigotica* were also indicated by transcriptome annotation. The metabolic profile of the flavonoids verified the catalytic action of putative FLS, F3′H, OMT, and related UGTs. On the other hand, some expected pathways as secologanin and isoflavonoids were not identified in *I. indigotica* transcriptome. The lack of these synthetic genes might indicate the absence of these metabolites in *I. indigotica*. Meanwhile, the low level of transcription was another possibility. Therefore, only the genome wide analysis could draw a full description of synthetic pathways.

### Co-expression analysis for the prediction of flavonoid composition in *I. indigotica*

The gene co-expression network models coordinated gene expression across the transcriptomic profile, which found a wide variety of applications in (systems) biology [41]. The constructed network demonstrated the signal pathways [42], transcriptional regulating network [43], and function of genes [27] in plant. In this paper, the co-expression analysis of *I. indigotica* UGTs based on the expression profile of homologous *Arabidopsis* genes was utilized for the functional prediction. The integration of sequence similarity and gene co-expression profiles allows the identification of conserved co-expression clusters among multiple plant species (so-called ‘comparative co-expression’) [44,45]. However, transcriptional analysis based on conversed expression of across species could only permit limited expression value to identify functional properties [46]. The application of next-generation sequencing to quantify plant transcriptional profile will generate new opportunities to study metabolism of *I. indigotica*. Base on the transcriptome annotation, the transcription profile of *I. indigotica* was able to establish. The co-expression network models would provide more precise and global insights into of secondary metabolites in *I. indigotica*.

In summary, *I. indigotica* is a suitable medicinal herbal model for investigating indole alkaloids, terpenoid and phenylpropanoids biosynthesis, but without genome-scale information. RNA sequencing makes it possible to carry out some “High-flux” analysis in *I. indigotica*. Here, the transcriptome annotation presents the most abundant genetic resource concerning *I. indigotica* to date. It will serve as the foundation for other functional genomic research efforts and provide the basis for improving the production of active compound through genetic engineering.

## Abbreviations

4CL: 4-coumarate; CoA: Ligase; DL7H: 7-deoxyloganin 7-hydroxylase; AACT: Acetyl-CoA C-acetyltransferase; ABP: Diamina oxidase; C4H: Cinnamate 4-hydroxylase; CAD: Cinnamyl alcohol dehydrogenase; CCoAoMT: Caffeoyl-CoA O-methyltransferase; CCR: Cinnamoyl-CoA reductase; CHI: Chalconeisomerase; CHS: Chalcone synthase; CMK: 4-(cytidine 5′-diphospho)-2-C-methyl-D-erythritol kinase; CPY: Cytochrome P450s; DDC: Aromatic amino acid decarboxylase; DIR: Dirgent; DMAPP: Dimethylallyldiphosphate; DXS: 1-deoxy-D-xylulose 5-phosphate synthase; DXR: 1-deoxy-D-xylulose 5-phosphate reductoisomerase; EIC: Extracted ion chromatogram; F3′H: Flavonoid 3′ hydroxylase; FDPS: Farnesyldiphosphate synthase; FLS: Flavonol synthase; FS II: Flavone synthase; G10H: Geraniol10-hydroxylase; GGPPS: Geranylgeranyldiphosphate synthase; GO: Gene ontology; GPPS: Geranyl pyrophosphate synthase; HCT: *p*-hydroxycinnamoyl-CoA shikimate/quinatehydroxycinnamoyltransferase; HDR: Hydroxymethylglutaryl-CoA reductase; HDS: 4-hydroxy-3-methylbut-2-enyl diphosphatereductase; HMGR: Hydroxymethylglutaryl-CoA reductase; HMGS: Hydroxylmethylglutaryl-CoA synthase; IAMT: Methyl indole-3-acetate methyltransferase; IFS: Isoflavon synthase; IPDC: Indolepyruvate decarboxylase; IPP: Isopentenyldiphosphate; IUGT: Indoxyl-UDPG-glucosyltranase; KEGG: Kyotoencyclopedia of genes and genomes; MCT: 2-C-methyl-D-erythritol 4-phosphate cytidylyltransferase; MDC: Mevalonate pyrophosphate decarboxylase; MDPS: Monoterpenyl-diphosphatase; MDS: 2-C-methyl-D-erythritol 2,4-cyclodiphosphate synthase; MeJA: Methyl jasmonate; MK: Mevalonate kinase; OMT: O-methyltransferase; ORF: Open reading frame; PAL: Phenylalanine ammonia-lyase; PHHA: Aromatic amino acid hydroxylase; PLR: Pinoresinol lariciresinol reductase; PMK: 5-phosphomevalonate kinase; RT-qPCR: Real-time quantitative PCR; SDH: Secoisolariciresinol dehydrogenase; STS: Stilbene synthase; TAL: Tyrosine ammonia-lyase; TCM: Traditional Chinese medicine; UGT: UDP-dependent glycosyltransferases; TPP: Tryptophanase; YUCCA: YUCCA family monooxygenase.

## Competing interests

The authors declare that they have no competing interests.

## Authors’ contributions

JC conceived the study, built the cDNA libraries, performed 454 sequencing, participated in the data analysis and drafted the manuscript. LZ performed most of the data analysis. QL carried out the annotation of unigenes. XD, RC, and SG participated in the chemical screening and data analysis. XZ performed the qRT-PCR analysis. LS contributed to the co-expression analysis. WC initiated the project, designed the study and participated in study coordination. All authors read and approved the final manuscript.

## Supplementary Material

Additional file 1 **Table S1.** The compounds isolated from *Isatis*. Click here for file

Additional file 2 Primers used in this study. Click here for file

Additional file 3 Queries used in co-expression analysis. Click here for file

Additional file 4 The length distribution of sequencing length of isogenes. Click here for file

Additional file 5 **Table S2.** The comparison of assembled results of combined reads and 454 reads. Click here for file

Additional file 6 **Gene Ontology (GO) functional annotations of *I. indigotica *** **isogenes.** Click here for file

Additional file 7 **COG functional annotations of *I. indigotica *** **isogenes.** Click here for file

Additional file 8 **KEGG annotation of *I. indigotica *** **transcriptome.** Click here for file

Additional file 9 **Table S3.** Information of 10 phenlypropanoid components detected in MeJA treated *I. indigotica* hariy roots. Click here for file

Additional file 10 The ECI profiles of flavonoids. Click here for file

Additional file 11 **The contribution of 41 UDP-dependent glycosyltransferase families in *I. indigotica***. Click here for file
