# Supplementary material for: Biosynthesis of the active compounds of Isatis indigotica based on transcriptome sequencing and metabolites profiling
Source: BMC Genomics. 2013 Dec 5;14:857. doi: 10.1186/1471-2164-14-857 (PMC3890716; doi:10.1186/1471-2164-14-857)
Supplement: Additional file 1 — Table S1. The compounds isolated from Isatis. [file 1471-2164-14-857-S1.docx]

| **Indole alkaloids** | | **Lignans** | **Others** |
| --- | --- | --- | --- |
| 1. **Indirubin**, C_16_H_10_N_2_O_2_  2.**Indigo**, C_16_H_10_N_2_O_2_  3. **Isoindigo**, C_16_H_10_N_2_O_2_  4. **Indoxyl**, C_8_H_7_N_O_  5. **5-Hydroxyoxindole**, C_8_H_7_NO_2_  6. **Bisindigotin**, C_32_H_18_N_4_O_2_  7. **Tryptanthrin**, C_15_H_8_N_2_O_2_  8. **N-formyl anthranilic acid**, C8H7NO3  9. **(2Z)-2-(1H-indol-3-ylmethylidene)-1,2-dihydro-3H-indol-3-one**, C_17_H_12_N_2_O  10. **3-(2′-Carboxyphenyl)-quinazolin-4-one**,  C_15_H_12_N_2_O_3_  11. **(E)-3-(3′,5′-dimethoxy-4′-hydroxy-benzylidene)-2-indolinone**, C_15_H_12_N_2_O_3_  12. **Indican**, C_14_H_17_NO_6_  13. **Glucobrassicin**, C_16_H_20_N_2_O_9_S_2_  14. **Indole-3-acetonotrile**, C_10_H_9_NO_3_  15. **Indole-3-acetic acid**, C_10_H_9_NO_2_  16. **Indole-3-carboxyaldehyd**, C_9_H_7_NO  17. **1-methoxyidole-3-carboxaldehyde**, C_11_H_11_NO_2_  18. **Indole-3-methyl acetate,** C_11_H_11_NO_2_  19. **Indole-3-carboxylic acid,** C_9_H_7_NO_2_  20. **Indole-3-acetonitrule-4-methoxy-2-C-β-D-glucopyraonoside**, C_17_H_20_N_2_O_5_  21. **N-methoxy-indole-3-acetonitrile-2-C-β-D-glucopyranoside,** C_17_H_20_N_2_O_5_  22.**2,3-dihydro-4-hydroxy-2-oxo-1H-indole-3-acetonitrile**, C_10_H_7_N_2_O_2_  23. **1H-indole-3-carboxylic acid**, C_9_H_7_NO_2_ | 24. **indole-3-methyl acetate**, C_11_H_11_NO_2_  25. **2,5-dihydroxy-indole**, C_8_H_7_NO_2_  26. **2,3-dihydro-4-hydroxy-2-oxo-indole-3- Acetonitrile**,C_10_H_8_N_2_0_2_  27**. Isaidigodione,** C_23_H_22_N_2_O_5_  28. **(−)-(R)-2-(3-Cyanomethyl-4-methoxy-1H-indol- 7-yl)-2-(1H-indol-3-yl)**  **acetonitrile**, C_21_H_16_N_4_O  29. **(−)-(R)-2-(3-Cyanomethyl-4-methoxy-1H-indol-7-yl)-2-(4-methoxy-1H-indol-3-yl)**  **acetonitrile**, C_22_H_18_N_4_O_2_  30.**(+)-(S)-2-{7-[1-(4-Hydroxyphenyl)ethyl]-4-methoxy-1H-indol-3-yl}acetonitril**e,  C_19_H_18_N_2_O_2_  31. **3-Hydroxy-2H-pyrrolo[2,3-b]indolo[5,5a,6-b,a] quinazoline-9- (8H),7′-dion**e,  C_17_H_12_N_2_O_3_  32**. Methyl 2-(4-oxo-1,4-dihydroquinoline-3-carboxamido) Benzoate**, C_18_H_14_N_2_O_4_  33. **6-Hydroxy-4-(5-hydroxymethylfuran-2-yl)- quinolin-2(1H)-one**, C_14_H_11_NO_4_  34. **(Z)-2-(1H-Indol-3-ylmethyldene)-1,2-dihydro-3H-indol-3-one**, C_17_H_12_N_2_O  35. **(+)-(R)-2-Oxo-1,2,3,4-tetrahydroquinoline-4-Carboxamide**, C_10_H_10_N_2_O_2_  36. **(+)-(R)-2-Methyl-7,8-dihydropyrano[4,3-b][1,4] oxazine-3,5- (2H,4H)-dione**,  C_8_H_9_NO_4_  37. **(+)-(S)-2-(3,4-Dihydroxy-2-oxoindolin-3-yl) Acetonitrile**, C_10_H_8_N_2_O_3_  38 **(−)-(R)-2-(4-Hydroxy-2-oxoindolin-3-yl) Acetonitrile**, C_10_H_8_N_2_O_2_  39. **(E)-2-(4-Hydroxy-2-oxoindolin-3-ylidene)acetonitrile**, C_10_H_6_N_2_O_2_  40. **(+)-(S)-2-(3-Hydroxy-4-methoxy-2-oxoindolin-3-yl) Acetonitrile**, C_11_H_10_N_2_O_3_  41. **(−)-(S)-2-(3-Hydroxy-2-oxoindolin-3-yl)acetamide,** C_10_H_10_N_2_O_3_  42. **(+)-(S)-2-(3-Hydroxy-4-methoxy-2-oxoindolin-3-yl) Acetamide**, C_10_H_12_N_2_O_4_  43. **(−)-(R)-2-(4-Hydroxy-2-oxoindolin-3-yl)acetamide**, C_10_H_10_N_2_O_3_  44. **(±)-2-(2-Oxoindolin-3-yl)acetamide**, C_10_H_10_N_2_O_2_  45. **9α,13α,-Dihydroxylisopropylidenylisatisine A**, C_25_H_22_N_2_O_6_  46. **Isatisine A**, C_22_H_18_N_2_O_6_ | 47. **Lariciresinol** C_20_H_24_O_6_ | 68.**Hypoxanthine**, C_5_H_4_N_4_O  69.**Uridine**, C_9_H_12_N_2_O_6_  70.**Adenosine**, C_10_H_13_N_5_O_4_  71.**Guanosine**, C_10_H_13_N_5_O_5_  72.**Progoitrin**, C_11_H_19_NO_10_S_2_  73.**Epiprogoitrin**, C_5_H_7_NOS  74.**R,S-goitrin**, C_11_H_19_NO_10_S_2_  75. **Gluconapin**, C_11_H_19_NO_9_S_2_  76.**Sinalbin**, C_14_H_19_NO_10_S_2_ |
|  |  | 48. **Secoisolariciresinol** C_20_H_26_O_6_  49. **Isolariciresin** C_20_H_24_O_6_  50. **Matairesnol** C_20_H_22_O_6_  51. **Pinoresinol** C_20_H_22_O_6_  52**.Lariciresinol-4,4′-bis-O-β-D-glucopyranoside**  C_32_H_44_O_16_  53**.Lariciresinol-4′-bis-O-β-D-glucopyranoside**  C_26_H_34_O_11_  54.**Lariciresinol-9-bis-O-β-D-glucopyranoside**  C_26_H_34_O_11_ |  |
|  |  |  | 77.**Sinigrin**, C_10_H_17_NO_9_ |
|  |  |  | 78.**(all-E)-Lutein**, C_40_H_56_O_2_  79.**(9Z)-Lutein**, C_40_H_56_O_2_ |
|  |  | **Flavonoids** |  |
|  |  | 55**. Vicenin-2,** C_27_H_30_O_15_  56. **Stellarin-2,** C_28_H_32_O_16_  57**. Saponarin** ,C_27_H_30_O_15_  58. **Isoorientin,** C_21_H_20_O_11_  59. **Isoorientin 3′′-O-glucopyranoside,** C_27_H_30_O_16_  60. **Isovitexin,** C_21_H_20_O_10_  61. **Isovitexin 3′′-O-glucopyranoside,** C_27_H_30_O_15_  62. **Isovitexin 6′′-O-glucopyronoside,** C_27_H_30_O_15_  63. **Isoscoparin,** C_22_H_22_O1_1_  64. **Isoscoparin 3′′-O-glucopyranoside,** C_28_H_32_O_15_ |  |
|  |  |  | **Reference** |
|  |  |  | **Compounds** 1-16，49-51, 76-77, (1, 2, 3) ; **Compounds** 19-27, (2); **Compounds** 31-49, Chen, *et al*., 2012; **Compounds** 47, 52, 53，Chen, *et al*., 2005; **Compounds** 54,70, Zuo *et al*., 2007; **Compounds** 55-64, deng *et al*., 2008; **Compounds** 55-74, 79, He, *et al*., 2005; **Compounds** 75-78,Shi *et a*l., 2012. |
|  |  | **Terpenoids** |  |
|  |  | 65. **β-sitosterol**, C_29_H_50_O  66.**γ- sitosterol,** C_29_H_50_O  67. **Daucosterol,** C_35_H_60_O_6_ |  |

**Additional file 1: Table S1** The compounds isolated from Isatis.

**Reference**

Mohn, T., Plitzko, I., and Hamburger, M. 2009, A comprehensive metabolite profiling of *Isatis tinctoria* leaf extracts. *Phytochemistry*, 70, 924–934.

Chen, M. H., Gan, L. S., Lin, S., Wang, X. L., Li, Y. H., Zhu, C. G., Wang, Y. N., Jiang, B. Y., Jiang, J. D., Yang, Y. C., and Shi, J. G. 2012, Alkaloids from the Root of *Isatis indigotica*. *J. Nat. Prod.*, 75, 1167−1176.

He, L. W., Li, X., and Chen, J. W. 2005, Research progress of antiviral active components of *Radix Isatidis*. *Inform. Trad. Chin. Med.*, 22, 37-40.

Deng, X. Y., Gao, G. H., Zheng, S. N., and Li. F. M. 2008, Qualitative and quantitative analysis of flavonoids in the leaves of *Isatis indigatica* Fort. by ultra-performance liquid chromatography with PDA and electrospray ionization tandem mass spectrometry detection. *J. Pharm. Biomed. Anal.*, 48, 562-567.

Li, B., Chen, W. S., Zhao, Y., Zhang, H. M., Dong, J. X., and Qiao, C. Z. 2005, Phenylpropanoids isolated from tetraploid roots of *Isatis indigotica*. *Chin. Trad. Herbal Drugs*, 36, 326-328 .

Liu, H. L., Wu, L. J., Li, H., and Wang, J. 2002, Study on the chemical constituents of *Isatis indigotica* Fort. *J. Shenyang Pharm. Univ.*, 19, 93-95.

Liu, J. F., Jiang, Z.Y., Wang, R. R., Zheng, Y.T., Chen, J.J., Zhang, X. M., and Ma,Y. B. 2007, Isatisine A, a novel alkaloid with an unprecedented skeleton from leaves of *Isatis indigotica. Org. Lett.*, 9, 4127-4129.

Liu, Y. H., Qin, G.W., Ding, S. P, and Wu, X. Y. 2002, Studies on chemical constituents in root of *Isatis indigotica* III. *Chin. Trad. Herbal Drugs*, 33, 97-99.

Zhou, L., Li, J. B., Xu, J., Yang, J. Z., Zhang, D. M, and Tong, Y. L. 2007, Studies on chemical constituents in root of *Isatis i ndigotica*. *China J. Chin. Materia Medica*., 32, 688-691.
