# Supplementary material for: Biosynthesis of the active compounds of Isatis indigotica based on transcriptome sequencing and metabolites profiling
Source: BMC Genomics. 2013 Dec 5;14:857. doi: 10.1186/1471-2164-14-857 (PMC3890716; doi:10.1186/1471-2164-14-857)
Supplement: Additional file 5 — Table S2. The comparison of assembled results of combined reads and 454 reads. [file 1471-2164-14-857-S5.docx]

**Additional file 5: Table S2** The comparison of assembled results of combined reads and 454 reads.

|  | 454+Solexa | 454 |
| --- | --- | --- |
| Total genes | 24,642 | 13,405 |
| Total isogenes | 36,367 | 16,728 |
| Average length | 115.67 | 971.9 |
| Largest isogene | 9012 | 5679 |
| Smallest isogene | 351 | 62 |
