# Supplementary material for: Biosynthesis of the active compounds of Isatis indigotica based on transcriptome sequencing and metabolites profiling
Source: BMC Genomics. 2013 Dec 5;14:857. doi: 10.1186/1471-2164-14-857 (PMC3890716; doi:10.1186/1471-2164-14-857)
Supplement: Additional file 9 — Table S3. Information of 10 phenlypropanoid components detected in MeJA treated I. indigotica hariy roots. [file 1471-2164-14-857-S9.docx]

**Additional file 9: Table S3** Information of 10 phenlypropanoid components detected in MeJA treated *I. indigotica* hariy roots.

| compounds | solvent | Formular | Mass | Product lon |
| --- | --- | --- | --- | --- |
| Coniferin | ethanol | C_16_H_22_O_8_ | 401.1 | 179 |
| Lariciresinol | ethanol | C_20_H_24_O_6_ | 359.2 | 329 |
| Secoisolariciresinol | ethanol | C_20_H_26_O_6_ | 361.1 | 164 |
| Mataireisnol | methanol | C_20_H_22_O_6_ | 357.1 | 121.9 |
| Pinoresinol | methanol | C_20_H_22_O_6_ | 357.1 | 151.1 |
| Coniferyl alcohol | methanol | C_10_H_12_O_3_ | 179 | 146 |
| Secoisolariciresinol diglucoside | methanol | C_32_H_46_O_16_ | 685.3 | 523.1 |
| Kaemferol | methanol | C_15_H_10_O_6_ | 286.24 | 117.0 |
| Quercetin | methanol | C_15_H_10_O_7_ | 302.24 | 151.1 |
| Isorhamnetin | methanol (5%DMSO) | C_16_H_12_O_7_ | 316.26 | 299.9 |
