# Supplementary material for: Biosynthesis of the active compounds of Isatis indigotica based on transcriptome sequencing and metabolites profiling
Source: BMC Genomics. 2013 Dec 5;14:857. doi: 10.1186/1471-2164-14-857 (PMC3890716; doi:10.1186/1471-2164-14-857)
Supplement: Additional file 10 — The ECI profiles of flavonoids. [file 1471-2164-14-857-S10.pdf]

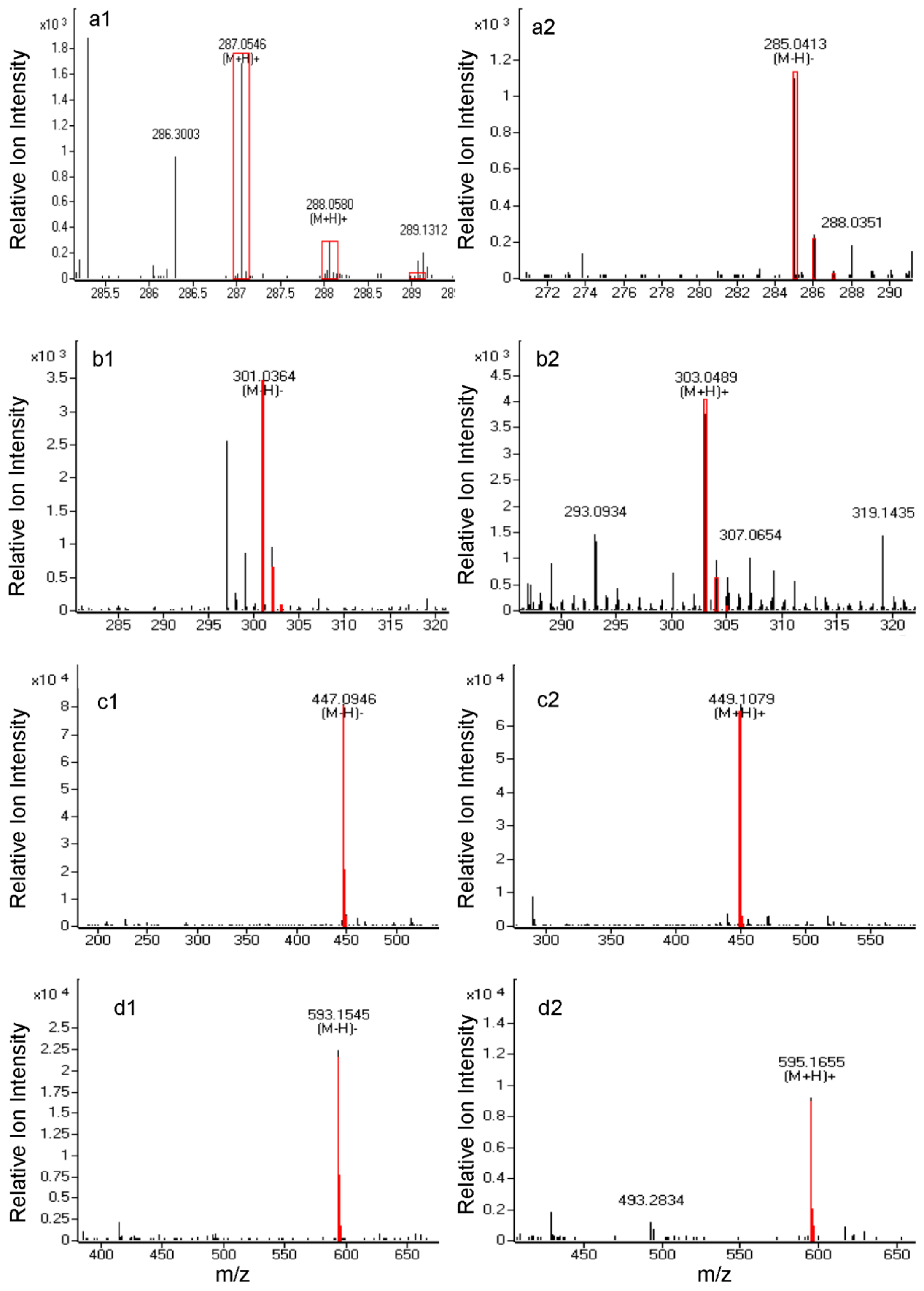

a1
x10 3
Relative Ion Intensity
287.0546
(M+H)+
286.3003
288.0580
(M+H)+
289.1312
a2
x10 3
285.0413
(M-H)-
288.0351
b1
x10 3
301.0364
(M-H)-
b2
x10 3
303.0489
(M+H)+
293.0934
307.0654
319.1435
c1
x10 4
447.0946
(M-H)-
c2
x10 4
449.1079
(M+H)+
d1
x10 4
593.1545
(M-H)-
m/z
d2
x10 4
595.1655
(M+H)+
493.2834
m/z

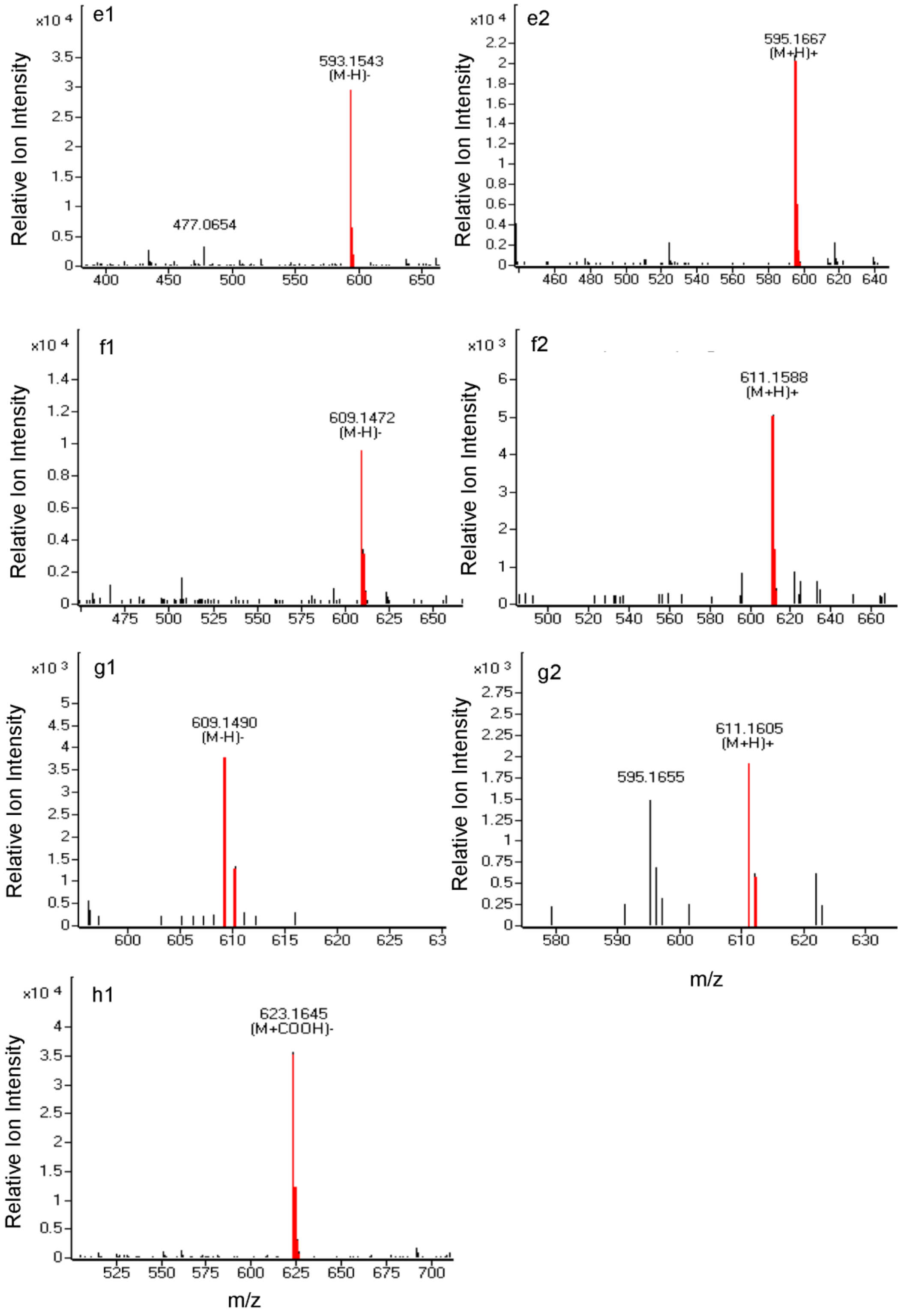

Additional file 10 The ECI profiles of flavonoids. a1, negative spectrum of kaempferol; a2, positive spectra of kaempferol; b1, negative spectrum of quercetin; b2, positive spectra of quercetin; c1, negative spectrum of kaempferol-3-O-glucoside; c2, kaempferol-3-O-glucoside; d1, negative spectrum of

kaempferol-3-O-rhamnoside-7-O-glucoside; d2, kaempferol-3-O-rhamnoside-7-O-glucoside; e1, negative spectrum of quercetin-3-O-rhamnoside-7-O-rhamnoside; e2, quercetin-3-O-rhamnoside-7-O-rhamnoside; f1, negative spectrum of quercetin-3-O-glucoside-7-O-rhamnoside; f2, quercetin-3-O-glucoside-7-O-rhamnoside; g1, negative spectrum of quercetin-3-O-rhamnoside-7-O-glucoside; g2, quercetin-3-O-rhamnoside-7-O-glucoside; h1, kaempferol-3-O-rhamnoside-7-O-rhamnoside.
