# Supplementary material for: Biosynthesis of the active compounds of Isatis indigotica based on transcriptome sequencing and metabolites profiling
Source: BMC Genomics. 2013 Dec 5;14:857. doi: 10.1186/1471-2164-14-857 (PMC3890716; doi:10.1186/1471-2164-14-857)
Supplement: Additional file 11 — The contribution of 41 UDP-dependent glycosyltransferase families in I. indigotica . [file 1471-2164-14-857-S11.pdf]

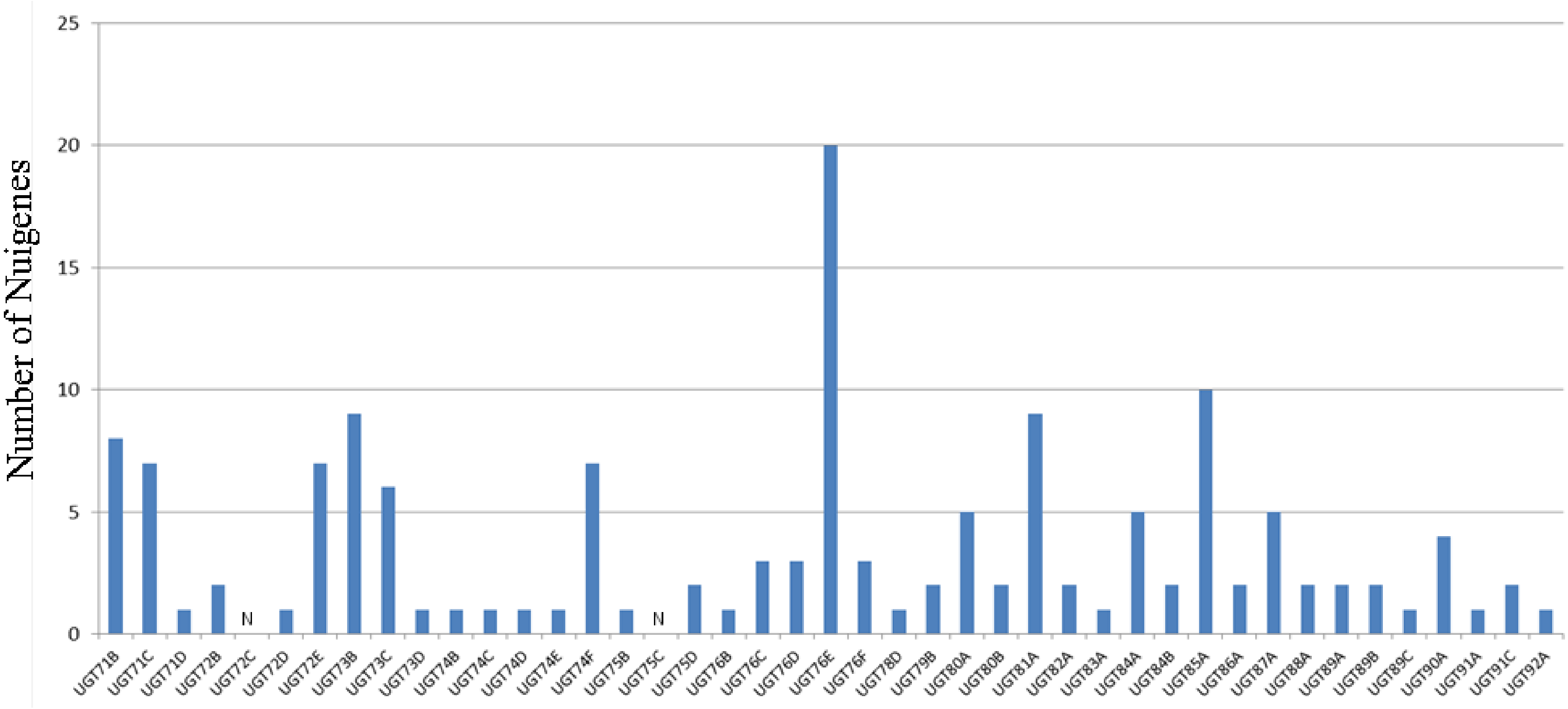

Additional file 11 The contribution of 41 UDP-dependent glycosyl transferases families in *I. indigotica*.UGT72C and UGT75C families were not found in *I. indigotica* transcriptome.
