# Supplementary material for: Biosynthesis of the active compounds of Isatis indigotica based on transcriptome sequencing and metabolites profiling
Source: BMC Genomics. 2013 Dec 5;14:857. doi: 10.1186/1471-2164-14-857 (PMC3890716; doi:10.1186/1471-2164-14-857)
Supplement: Additional file 6 — Gene Ontology (GO) functional annotations of I. indigotica isogenes. [file 1471-2164-14-857-S6.pdf]

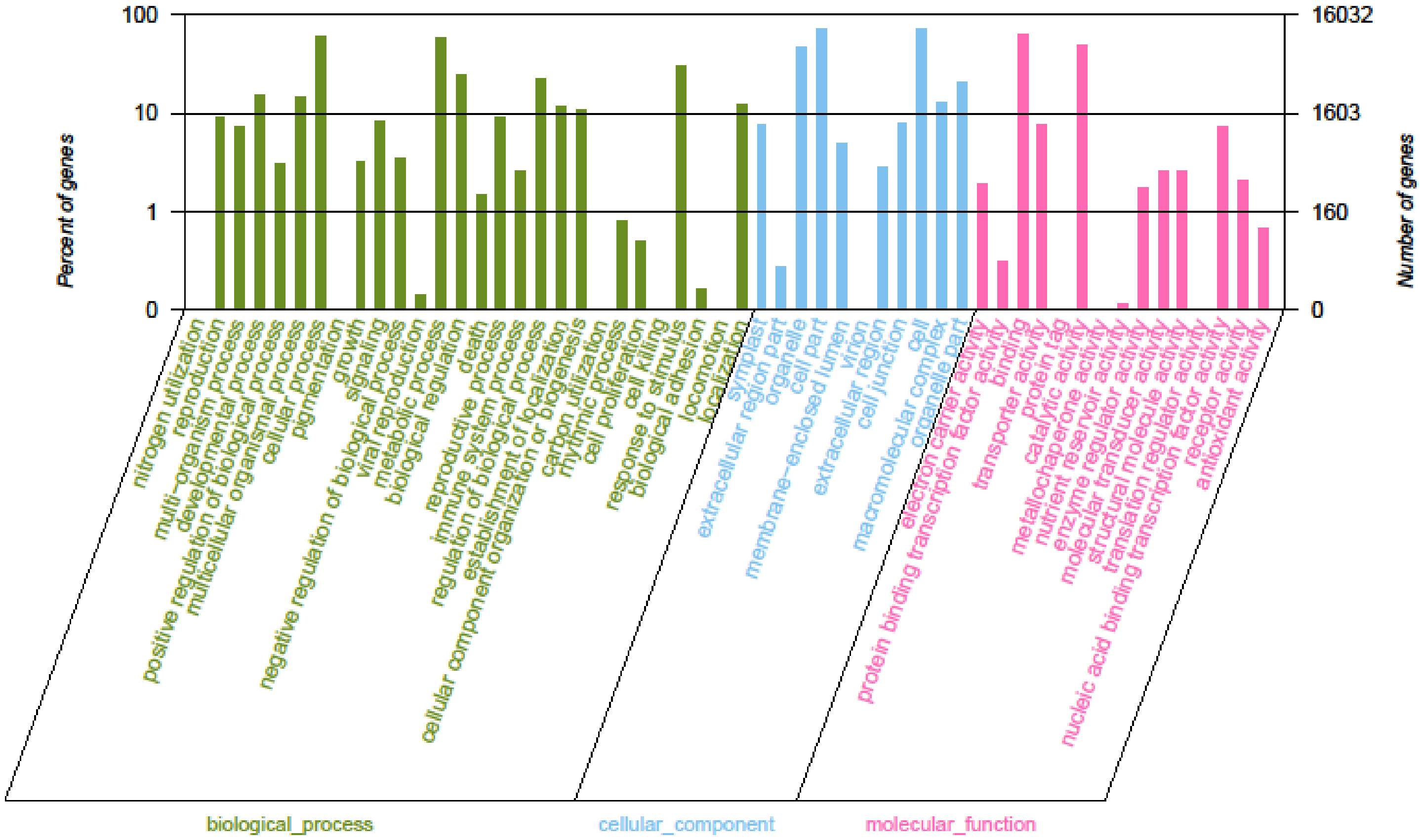

Additional file 6 Gene Ontology (GO) functional annotations of *I. indigotica* isogenes.
